# Infant anemia is associated with reduced TLR-stimulated cytokine responses and increased nasopharyngeal colonization with *Moxarella catarrhalis*

**DOI:** 10.1038/s41598-018-23264-y

**Published:** 2018-03-20

**Authors:** Sui-Ling Liao, Shih-Yun Hsu, Shen-Hao Lai, Shih-Hsiang Chen, Man-Chin Hua, Tsung-Chieh Yao, Li-Chen Chen, Ming-Han Tsai, Jing-Long Huang

**Affiliations:** 1 Community Medicine Research Center, Chang Gung Memorial Hospital at Keelung, Keelung, Taiwan; 2 Department of Pediatrics, Chang Gung Memorial Hospital at Keelung, Keelung, Taiwan; 3 Division of Pulmonology, Department of Pediatrics, Chang Gung Memorial Hospital and Chang Gung University, College of Medicine, Taoyuan, Taiwan; 4 Division of Oncology and Hematology, Department of Pediatrics, Chang Gung Memorial Hospital and Chang Gung University, College of Medicine, Taoyuan, Taiwan; 5 Division of Allergy, Asthma, and Rheumatology, Department of Pediatrics, Chang Gung Memorial Hospital and Chang Gung University, College of Medicine, Taoyuan, Taiwan

## Abstract

Anemia is a major public health problem in young children. Reports on the role of anemia on infectious diseases remained controversial. We aim to investigate the effect of anemia on innate immunity, nasopharyngeal bacterial colonization, and subsequent infectious outcome. Blood tests were examined at the age of 12 months. TLR-induced cytokine production was assessed by ELISA. Bacteria from nasopharyngeal specimens were identified with traditional culture. Clinical infectious diseases were followed yearly until 3 years of age. Result showed that of the 423 infants, 72 had hemoglobin level ≤ 11 g/dL, among which 55% had normal iron level. There was significant association between hemoglobin level and TLR1–2, and 4 induced IL-6 (p = 0.04, 0.02) and that of TLR4 stimulated TNF-α response (p = 0.04). Children with anemia had higher nasopharyngeal colonization with *Moxarella catarrhalis*. Clinical analysis did not show anemia to be associated with infectious morbidity. However, children who developed LRTIs had mean lower ferritin levels. We speculated that iron might be the key factor related to infectious morbidity. Thus, to investigate the role of anemia in infectious diseases, it is important to first consider the prevalence of iron deficit, since the incidence of iron deficiency-induced anemia may vary among different regions.

## Introduction

Anemia is a major public health problem that occurs more prevalently in young children and pregnant women. About 45% of preschool children are affected worldwide^1^. Measurement of hemoglobin level is the most commonly used indicator for anemia among all individuals. The causes of anemia may vary by age. Those occurring in early childhood include iron deficiency, concurrent viral or bacterial infection, blood loss due to trauma or gastrointestinal bleeding, disorders of hemoglobin structure (thalassemia, sickle cell disease, spherocytosis), enzyme defects (G6PD or pyruvate kinase deficiency), micronutrient deficiency such as zinc or copper, and rare causes such as neoplastic diseases, immune-mediated, parasitic infestations, or lead poisoning. Although iron deficiency remained to be the most common cause of anemia in young children, around 35–40% of children with anemia were associated with normal iron status^2–4^. Understanding the relative importance of anemia in infancy, including those without iron deficiency is necessary in order to assess its impact on children’s clinical health. The association between low hemoglobin level and pediatric infectious diseases, especially that of lower respiratory tract infection, has been reported by several studies, however the results remained debatable^5–10^. Childhood anemia at high altitude also posed as a risk factor for poorer outcomes in severe pneumonia^11^. Although animal and *in-vitro* tests have found iron to play an important role in human defense against infections, however, in developed countries, only around 50% of infant anemia was caused by iron deficiency, thus, the association between anemia, whatever the etiology, and human infectious morbidity remained to be further explored. In this study, we aimed to investigate if infant anemia (with or without iron-deficiency) was associated with altered innate immune cytokine response, modifications in nasopharyngeal bacterial colonization rate, and subsequent infectious outcome in young children.

## Results

### Subject and demographic data

**Figure 1:**
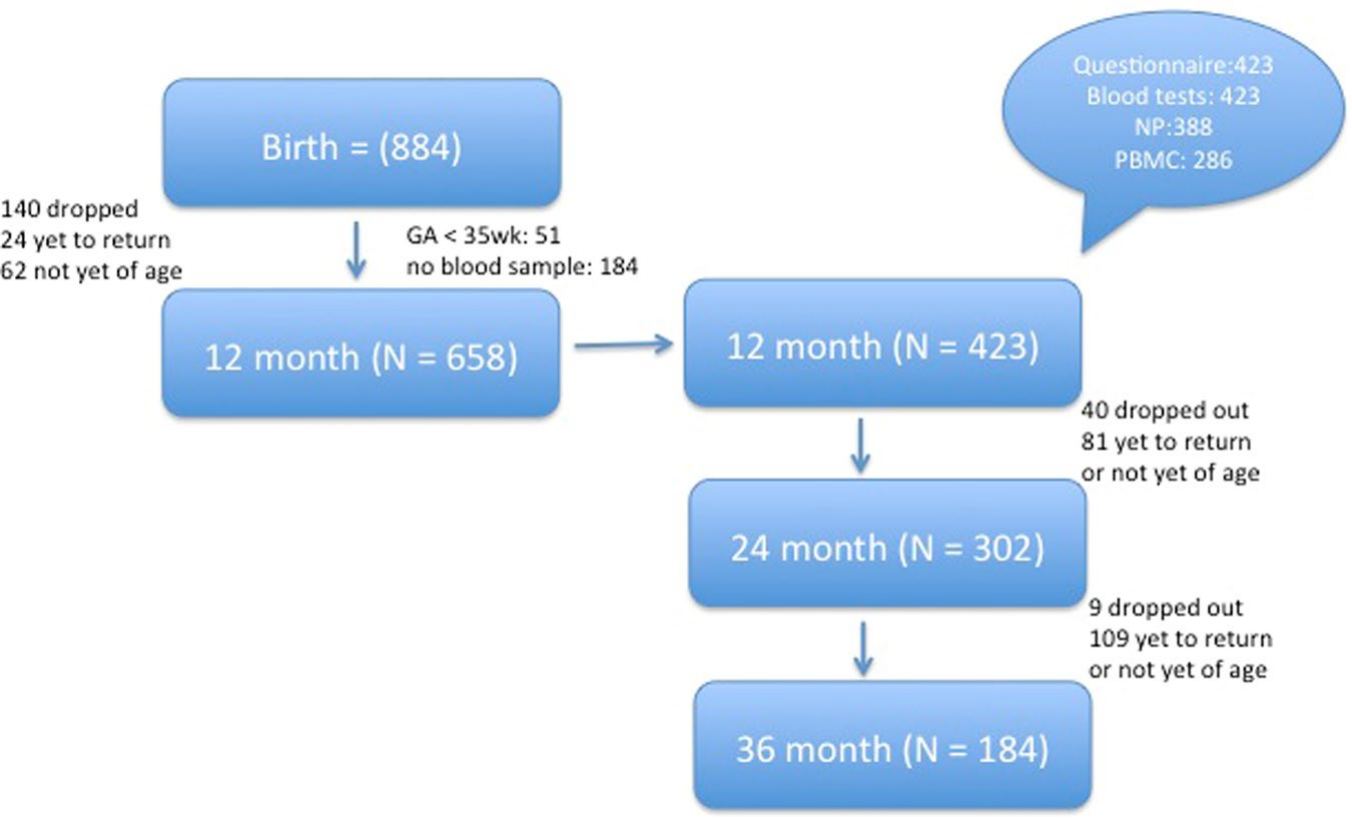
Flow chart of the birth cohort study: demonstrating the number of participants at each age period. 423 infants at the age of 12 months had complete questionnaire results and blood tests (complete blood count), 388 had nasopharyngeal specimen (NP) for bacterial identification, and 286 infants had peripheral blood mononuclear cell (PBMC) available for TLR stimulation. Valid questionnaire information and medical records in regard to infectious diseases were available for 302 children at the age of 24 months and 184 at the age of 36 months.

A total of 884 infants were enrolled in our research program since birth. Children without sufficient blood samples, and those born prematurely (GA < 35 weeks) were excluded from this analysis. By the age of 1 year, 423 infants had blood test results and complete questionnaire data. Detailed number of participants and test samples is listed in Fig. 1. Characteristics of the infants with and without anemia are summarized in Table 1. Of the 423 infants, 72 infants (17%) had hemoglobin level ≤ 11 g/dL. There were slightly more male infants in the anemic group. Infants with anemia had significantly longer duration of breastfeeding, a mean lower serum ferritin level, and smaller MCV. 45% of the infants with anemia had serum ferritin level of less than 10 ng/mL (meeting the criteria of iron-deficiency), 8 anemic infants (11%) were either diagnosed with or had a family history of thalassemia. 3 infants with anemia (4.2%) has G6PD deficiency, but did not have hemolysis during blood testing. Although zinc deficiency is a risk factor for anemia, and rivals iron deficiency as a major contributor to overall anemia in developing countries^12,13^, none of the anemic infants in our population had serum zinc level of <60 ng/dL (meeting the criteria of zinc deficiency). Serum zinc level was similar between children with or without anemia.

**Table 1:** Demographic characteristic of infants with Hb below and above 11 g/dL.

Characteristics	Hb ≤ 11 g/dL n (%)	Hb > 11 g/dL n (%)	p
Sex (male)	48 (67.1)	189 (53.8)	0.05
Gestational Age	38.2 ± 1	38.4 ± 1	0.31
Birth Body Weight (g)	3101 ± 379	3120 ± 432	0.75
Mode of delivery			
NSD	45 (62.7)	218 (62.1)	1.00
Duration of BF (mo)	8.8 ± 5	3.6 ± 4	<0.01
Time of solid food (m/o)	5.2 ± 0.8	5.1 ± 0.9	0.20
Body Height 1 y/o (cm)	74.8 ± 2.7	75.1 ± 3.2	0.34
Father allergy	28 (38.9)	110 (31.3)	0.21
Mother allergy	23 (31.9)	117 (33.3)	0.82
MCV	67.8 ± 8	75.5 ± 4	<0.01
Ferritin (ng/mL)	16.4 ± 11.2	40.6 ± 35.8	<0.01
Zinc (ng/mL)	81.2. ± 11.8	79.6 ± 12.7	0.58
Maternal Education			0.51
Primary or secondary	2 (2.9)	8 (2.3)	
High school	18 (25.0)	87 (24.8)	
College or above	52 (72.1)	256 (72.9)	

Total number of Hb ≤ 11 g/dL: 72

Total number of Hb > 11 g/dL: 351

NSD: natural spontaneous delivery

BF: breastfeeding in months

Age in months at the time of solid food introduction

### Association between hemoglobin level and toll-like receptor stimulated cytokine response

Because the distribution of most cytokine levels was highly skewed (data not shown), we used natural log-transformed cytokine levels for correlation analysis. The result showed a significant positive correlation between hemoglobin level and TLR4 –induced TNF-α and IL-6 response. (aOR: 0.26; 95% CI: 0.02–0.5 for TNF-α, and aOR: 0.43; 95% CI: 0.07–0.78 for IL-6). There was a possible correlation between hemoglobin level and TLR1–2 induced IL-6 response after adjusting for confounding factors (aOR: 0.21; 95% CI: 0.005–0.41). Our result did not show hemoglobin level to be associated with any of the TLR stimulated IL-10 responses, an anti-inflammatory cytokine (Table 2).

**Table 2:** Association between hemoglobin level and toll-like receptor-induced cytokine response.

	Univariate analysis		Multiviariate analysis	
	β (95% CI)	p	β (95% CI)	p
***TLR1-2***				
TNF-α	0.02 (−0.09, 0.14)	0.70	0.03 (−0.08, 0.14)	0.62
IL-6	0.16 (−0.04, 0.36)	0.12	0.21 (0.005, 0.41)	0.04
IL-10	0.11 (−0.03, 0.26)	0.13	0.08 (−0.07, 0.24)	0.06
***TLR3***				
TNF-α	−0.001 (−0.06, 0.05)	0.96	0.01 (−0.05, 0.12)	0.68
IL-6	−0.01 (−0.11, 0.09)	0.88	−0.01 (−0.11, 0.10)	0.91
IL-10	0.02 (−0.07, 0.11)	0.63	−0.02 (−0.12, 0.08)	0.69
***TLR4***				
TNF-α	0.22 (−0.02, 0.45)	0.07	0.26 (0.02, 0.50)	0.04
IL-6	0.34 (0.003, 0.67)	0.05	0.43 (0.07, 0.78)	0.02
IL-10	0.10 (−0.05, 0.25)	0.21	0.08 (−0.09, 0.25)	0.36
***TLR7-8***				
TNF-α	0.15 (−0.04, 0.34)	0.12	0.17 (−0.03, 0.36)	0.09
IL-6	0.10 (−0.15, 0.35)	0.45	0.20 (−0.06, 0.46)	0.13
IL-10	0.01 (−0.11, 0.13)	0.84	0.02 (−0.13, 0.16)	0.81
***PHA***				
TNF-α	0.05 (−0.05, 0.14)	0.32	0.08 (−0.004, 0.16)	0.06
IL-6	−0.04 (−0.19, 0.11)	0.60	−0.03(−0.17, 0.11)	0.66
IL-10	−0.05 (−0.17, 0.08)	0.47	0.01 (−0.12, 0.14)	0.85

Adjusted for gestational age, gender, body weight and height at 1 year, mode of delivery, breastfeeding duration, parental allergy, and maternal education.

### Association between hemoglobin level and nasopharyngeal bacterial colonization

The study from Pichichero had shown a significant correlation between serum inflammatory cytokine production and asymptomatic nasopharygeal bacterial colonization^14^. Since we had observed a reduced TNF-α and IL-6 response to TLR1-2 and TLR4 stimulation in anemic children, we had then proceeded to investigate whether the result of decreased pro-inflammatory cytokines would lead to modifications in nasopharyngeal bacterial colonization. The results showed that although *S. aureus* was the most common organism found in the airway, the rate of colonization was relatively similar between children with or without anemia. However, infants with anemia appeared to have higher prevalence of nasopharyngeal colonization with *M. catarrhalis*, with 12.7% positive colonization rate in the anemic children vs. 4.7% in non-anemic children (p = 0.01) (Table 3) The correlation still remained significant when using hemoglobin level as continuous variables and adjusting for confounding factors (Table 4).

**Table 3:** Prevalence of bacteria colonization in children with and without anemia.

	Hb < 11 g/dL n (%)	Hb > 11 g/dL n (%)	p
Negative	50 (70.4)	255 (80.4)	0.06
*S. aureus*	6 (8.5)	29 (9.2)	0.85
*S. pneumoniae*	2 (2.8)	8 (2.5)	0.89
*M. catarrhalis*	9 (12.7)	15 (4.7)	0.01
*H. influenzae*	3 (4.2)	10 (3.2)	0.65
Other	1 (1.4)	0	N/A

Total number of nasopharyngeal specimen: Hb < 11 g/dl: 71 and Hb > 11 g/dl: 317 *S. aureus*: *Staphylococcus aureus* (includes both methicillin-resistant and methicillin-

sensitive *Staphylococcus aureus*).

*S. pneumoniae*: *Streptococcus pneumoniae*

*M. catarrhalis*: *Moraxella catarrhalis*.

*H. influenzae*: *Hemophilus influenzae*.

Other: Acinetobacter species.

Analysis by Chi-square test.

**Table 4:** Relationship between nasopharygeal colonization and Hb level.

Organism	Univariate analysis OR (95% CI)	p	Multivariate analysis OR (95% CI)	p
*S. aureus*	1.00 (0.74~1.36)	0.99	0.88 (0.64~1.21)	0.42
*S. pneumoniae*	0.97 (0.55~1.71)	0.91	1.21 (0.60~2.44)	0.59
*M. catarrhalis*	0.70 (0.50~0.99)	0.042	0.69 (0.47~0.99)	0.047
*H. influenzae*	0.71 (0.47~1.09)	0.11	0.67 (0.45~1.09)	0.11
Others	0.46 (0.14~1.48)	0.19	0.43 (0.13~1.48)	0.18

Adjusted for gestational age, breastfeeding duration, gender, and mode of delivery.

### Association between hemoglobin level and the risk of infection during early childhood

Since we had found hemoglobin level to be associated with alterations in TLR induced pro-inflammatory cytokine response and increased nasopharyngeal colonization with *M. catarrhalis*, we had then proceeded to investigate whether these effects might have potential impact on infectious outcome. By 3 years of age, complete medical records and questionnaires were available for 423 infants at 1- year, 302 infants at 2- years, and 184 infants at 3- years of age. The result did not show significant correlation between hemoglobin level and the incidence of infectious diseases such as croup, low respiratory tract infections (LTRIs), acute otitis media, urinary tract infection, and acute gastroenteritis during the first three years of life. Anemia also did not increase the risk for hospitalization during early life (Table 5).

**Table 5:** Association between Hb level at 12 months and infectious diseases during early childhood.

	Crude OR (95% CI)	p	Adjusted OR (95% CI)	p
***1 year old***				
LRTI	0.71 (0.34–1.45)	0.34	0.79 (0.26–2.40)	0.68
Croup	0.55 (0.11–2.77)	0.47	0.22 (0.02–2.06)	0.18
AOM	0.71 (0.43–1.12)	0.18	0.89 (0.61–1.31)	0.41
AGE	1.10 (0.69–1.72)	0.71	0.84(0.48–1.49)	0.55
UTI	0.95 (0.66–1.37)	0.79	0.71(0.43–1.15)	0.16
Hospital	0.66 (0.31–1.42)	0.29	0.44 (0.14–1.37)	0.16
***2 years old***				
LRTI	1.07 (0.56–2.06)	0.83	1.03 (0.43–2.49)	0.95
Croup	0.89 (0.19–4.26)	0.89	0.54 (0.05–6.03)	0.62
AOM	1.04 (0.89–1.21)	0.63	4.12 (0.47–37.01)	0.20
AGE	1.04 (0.91–1.17)	0.60	1.60 (0.45–5.70)	0.47
UTI	1.03 (0.91–1.16)	0.69	1.45 (0.27–7.91)	0.66
Hospital	0.98 (0.48–1.97)	0.95	1.16 (0.37–3.62)	0.80
***3 years old***				
LRTI	2.02 (0.93–4.41)	0.08	2.05 (0.76–5.56)	0.16
Croup	0.59 (0.20–1.77)	0.35	1.20 (0.24–5.97)	0.82
AOM	0.94 (0.77–1.45)	0.54	1.01 (0.18–6.56)	0.92
AGE	1.04 (0.89–1.20)	0.64	1.94 (0.44–8.65)	0.38
UTI	0.95 (0.81–1.12)	0.57	2.53 (0.29–21.90)	0.40
Hospital	1.21 (0.52–2.84)	0.66	1.16 (0.37–3.62)	0.80

OR: odds ratio: adjusted for gender, mode of delivery, body weight and height, duration of breastfeeding, parental allergy, and maternal education

*18 children had received iron supplement after the age of 1 year, and was included in the analysis at the ages 2 and 3 years

LRTI: low respiratory tract infection (acute bronchiolitis and/or pneumonia)

AOM: acute otitis media

UTI: urinary tract infection

AGE: acute gastroenteritis

Hospital: hospitalization.

Due to the fact that ferritin was known to be an important immune-modulator, especially in the respiratory tract, serum ferritin levels were compared in children with or without LTRIs during their first year of life. The result showed that children who have had LRTIs during their infancy had mean lower ferritin level than those never suffered from low respiratory tract infections. Mean ferritin levels at age 1 year were 22.1 ± 11.9 ng/mL for children with ever having LRTIs, and 34.5 ± 21.5 ng/mL for those never suffered from any episodes of LTRIs (p = 0.03) (supplement).

## Discussion

Results from current study showed that infant anemia was associated with decreased pro-inflammatory cytokine responses to TLR1-2 and TLR4 stimulation, as well an increased prevalence of nasopharyngeal colonization with *M. catarrhalis*. However, despite such effect, anemia during infancy was not associated with subsequent infectious morbidity in childhood. Similar to our results, Broor *et al*. did not find anemia to be a risk factor for low respiratory tract infection in 521 children under the age of five years^15^. Harris *et al*. also did not find potential interaction between pneumonia hospitalization and anemia status, however, anemia increased the odds for pneumonia hospitalization if children were exposed under higher air pollution^7^. In addition, in a large prospective clinical trial, although provision of iron at 6 months of age was associated with decreased risk of anemia, however, the incidence of infectious diseases such as bloody diarrhea and acute respiratory illness were inadvertently increased following iron supplementation^16^. In contrast to these observations, several studies have concluded differently, indicating that anemia increased the odds of low respiratory tract infection by approximately 2 to 5 times^6,8,9^. However, results from these studies were difficult to ascertain, as most reports were of retrospective hospital-based studies that compared sick children with healthy controls. Acute infection or inflammation can decrease hemoglobin levels through hemolysis, reduction of iron absorption, inhibition of erythropoietin release, or side effects from medication^17–19^. Thus, casualty between anemia and increased risk of infectious diseases could not be established, since children could become anemic as a result of acute infection or repeated infections. In order to determine the cause and effect relationship between anemia and infectious morbidity, data from a prospective cohort study would yield more reliable results. Hence, we had examined hemoglobin level in 1-year-old healthy infants free from infection, and subsequently followed these children’s health status up to the age of 3 years. Our data demonstrated that anemia during infancy, whatever the etiology, was not a significant predictor for subsequent infectious diseases. Yet, in contrast to our observation, a prospective study conducted in southern Israel had suggested that anemia at 6 months of age was associated with increased rates of infection such as diarrhea, otitis media, and respiratory illness later at ages 7–18 months^10^. The reason for such association might be related to the high prevalence of iron deficiency. Although ferritin level was not measured, their report claimed that the majority of the causes for infant anemia in Israel was mainly due to iron –deficiency^20^. Iron has been shown to play an important role in the immune system. *In vitro* studies have shown iron deficiency to impair immune function by decreasing humoral immunity, lymphocyte cytokine production, and neutrophil phagocytic activity^21,22^. Results from our study had also shown that children who developed LRTIs had mean lower serum ferritin levels. We speculated that iron might be the key factor associated with infectious morbidity. Since about half of the anemic infants in our study had normal iron status, it might be the reason that we did not find anemia to be associated with clinical infections. It is possible that the controversy between anemia and infectious diseases might depend on the prevalence and severity of iron deficiency. In areas where iron deficiency is the major cause of anemia, the association with infectious diseases may be more evident. While in areas where iron deficiency is not the utmost cause, like our population, then the connection may not be as significant. Even though anemia is a poor predictor of iron deficiency, still, numerous studies refer anemia as a form of iron deficiency, thus leading to biased conclusions and controversies.

Our study had shown anemia to be associated with decreased pro-inflammatory cytokine response to TLR1-2 and TLR4 stimulation; however, it was not associated with clinical infection. The mechanism underlying this null association is yet not fully understood, but may be explained by the fact that since immune system is composed of multiple cells and variable signaling cascades that can also influence adaptive immunity, suppression of certain cytokines of the innate immune system may not have an overall effect on the disease outcome. Besides, very few children had hemoglobin level between 7.0–9.9 g/dL (meeting the definition of moderate anemia) and even fewer children had level of less than 7.0 g/dL (meeting the definition of severe anemia). Thus, connection between the severe form of anemia and infectious outcome could not be established. There is a probability that the effect of anemia on infectious morbidity may only be evident if hemoglobin is lower than certain threshold.

In addition, compared to children without anemia, we had observed an increased prevalence of nasopharygeal colonization with *M. catarrhalis* in children with lower hemoglobin level. However, it was not associated with clinical morbidity. Reports have indicated that although colonization by *H. influenzae* and *Streptococcus pneumoniae* in the airway were associated with higher frequencies of LRTIs, those colonized by *M. catarrhalis* or *S. aureus* were not correlated with clinical diseases of the respiratory tract. Thus, although our study had shown children with anemia to have higher rates of nasopharyngeal colonization with *M. catarrhalis*, it was not a risk factor for subsequent infection^23,24^.

The strength of this report is that as a prospective cohort study, we were able to monitor children’s clinical health after hemoglobin assessment, providing stronger evidence in determining the effect of infant anemia on subsequent infectious outcome. However, our study has several limitations. First, there were only 72 children with hemoglobin less than 11 g/dl, and the cause of anemia was undetermined in approximately 40% of the children. Thus, the statistical power might be reduced due to small population size. Second, due to limitations in the amount of blood drawn from small children, C-reactive protein level and other laboratory tools such as transferrin receptors were not examined in our study, thus, the true incidence of iron deficiency in our population remained uncertain. However, blood examination was not performed within 3 weeks of active infection, thus, the effect of infection on serum ferritin level was minimized.

In summary, anemia in infancy was associated with reduced pro-inflammatory cytokine response to TLR1-2 and TLR4 stimulation, as well an increased prevalence of nasopharyngeal colonization with *M. catarrhalis*. Although we found a negative association between anemia and subsequent infectious diseases in children, we believed that our results were able to elucidate some theoretical assumptions about the relationship between anemia and pediatric infections. In addition, in areas where iron deficiency is not the main cause of anemia, the effect of iron alone (not in the form of anemia) on pediatric infectious diseases might warrant further in-depth investigation.

## Methods

### Study Population

Data for this analysis came from an ongoing prospective birth cohort study called the PATCH (The Prediction of Allergy in Taiwanese Children). This study was approved by The Chang Gung Ethics Committee, and informed consent was obtained from the parents/legal guardians of the neonates. All methods were performed in accordance with the relevant guidelines and regulation. By the time of analysis, 884 neonates were enrolled in our research program, among which 658 infants remained in the study and reached the age of 12 months. After excluding premature infants with the gestational age of < 35 weeks, and those without blood samples (either due to refusal and/or difficulty in obtaining blood), a total of 423 infants were included in this study. A baseline questionnaire survey was conducted at birth to obtain information such as birth body weight and height, medical and obstetric history, and family history of blood diseases (thalassemia, sickle cell or G6PD deficiency). Children were examined by a study physician at ages 2, 4, 6, and 12 months, and every year thereafter. Standardized questionnaires on dietary practices, environmental factors, infectious and allergic diseases were obtained. Children were defined as ever having lower respiratory tract infection (bronchiolitis and/or pneumonia) if there was a diagnosis from a health care professional, and the infant had either been hospitalized or received medical treatment. Other infections such as otitis media, croup, infectious enteritis, and urinary tract infection were also obtained from medical records with physicians’ diagnosis. By the time of analysis, 423 infants had complete blood test results and questionnaire assessment at 1 year of age. Complete questionnaire survey and medical records were available for 302 children at the age of 2 years, and 184 children by 3 years of age (Fig. 1).

### Sample collection and blood Assay

Peripheral bloods were collected at the age of 12 months and sent to central clinical laboratory to test for complete CBC count, serum ferritin, and zinc level. Hemoglobin level was measured in blood samples by using the automatic blood cell counter. Anemia was defined as hemoglobin level ≤ 11 g/dl^25^. Serum ferritin level, which is directly proportional to the body’s iron stores, was measured by ADVIA Centaur Ferritin assay, a two-site sandwich immunoassay using direct chemiluminometric technology. To avoid the effect of infection on measured values, drawing of blood samples were delayed if the infant had any signs of infection (including fever, rhinorrhea, cough, diarrhea, or any other discomforts suspicious of infection) within 3 weeks of blood testing.

### Cell culture and TLR ligands stimulation

Mononuclear cells were isolated from peripheral blood and stimulated with TLR ligands. These ligands included synthetic bacterial lipoprotein (PAM3csk4) that is recognized by TLR1-2 at a concentration of 10 ug/ml; a synthetic analog of double stranded RNA for TLR3, using 10 ug/ml of poly(I:C) directly administered to the cells; a concentration of 20 ng/ml of ultra pure LPS for TLR4; and R848 (10 ug/ml), which activates via the TLR7/TLR8 signaling pathway (InvivoGen, San Diego, CA). As a positive control, cells were treated with the NF-kB activator phytohemagglutinin (Murex Pharmaceuticals) at 4 ug/ml in R10-FBS. To determine TLR responses, 3 × 10^5^ PBMCs in 100 ul R10-FBS were added to each of the medium or ligand (in duplicate) - containing wells and incubated at 37 °C for 20 h with 5% CO2. All assay preparations were performed using sterile technique in a laminar flow hood. The details of our experimental procedures have been published previously^26^.

### Measurement of cytokines

TNF-α, IL-10, and IL-6 levels in culture supernatants were determined by enzyme-linked immunosorbent assays according to the manufacturer’s instructions (ELISA; R&D systems, MN). The detection limits were 15.6 pg/mL for TNF-α, 3.12 pg/mL for IL-6, and 7.8 pg/mL for IL-10.

### Bacterial identification from nasopharyx

Nasopharyngeal specimens were obtained at 12 months of age through the nose with separate cotton-tipped swabs (Copan Swab Applicator, Copan Diagnostics Inc., Brescia, Italy). Samples were then transported to the microbiology laboratories within 2 hours of collection and cultured for bacteria with the use of standard methods for identification. Throat swab were performed only in children free of any respiratory symptoms for at least 3 weeks. Details of our experimental procedures have been published previously^27^.

### Statistical Methods

Multiple linear regression analysis was used to determine the relationship between hemoglobin level and TLR-induced cytokine response. Since cytokine levels were not normally distributed, values were logarithmically transformed as continuous variables in the statistical models. Association between hemoglobin level and binary outcomes (low respiratory tract infection, croup, acute otitis media, infectious enteritis, urinary tract infection, and hospitalization) were analyzed by using logistic regression. Student T test was used to compare serum ferritin level between infants with or without ever having LRTIs during infancy. Factors such as gestational age, birth body weight, mode of delivery, gender, parental history of allergy, maternal education, and exclusive breastfeeding for longer than 4 months were included in the multiple regression analysis to compensate confounders’ effects. Confounding effect for the relationship between hemoglobin level and infectious outcome included body weight and height at 12 months to avoid the effect of malnutrition on infectious tendency. Following laboratory results tested by 1 year of age, 18 anemic children had received iron supplements (ferrous sulfate at 1 mg/kg/d for 3–6 months), thus was included in subsequent outcome analysis as a possible confounding factor. All statistical analysis was carried out using IBM SPSS Statistics Version 20 (Armonk, NY). The datasets analyzed during the current study are available from the corresponding author on reasonable request.

## Electronic supplementary material

Supplementary Information
