# Supplementary material for: Infant anemia is associated with reduced TLR-stimulated cytokine responses and increased nasopharyngeal colonization with Moxarella catarrhalis
Source: Sci Rep. 2018 Mar 20;8:4897. doi: 10.1038/s41598-018-23264-y (PMC5861055; doi:10.1038/s41598-018-23264-y)
Supplement: Supplementary file 1 — Supplementary Information [file 41598_2018_23264_MOESM1_ESM.docx]

Infant anemia is associated with reduced TLR-stimulated cytokine responses and increased nasopharyngeal colonization with *Moxarella catarrhalis*

Sui-Ling Liao^1,2^, Shih-Yun Hsu^1,2^, Shen-Hao Lai^1,3^, Shih-Hsiang Chen^4^, Man-Chin Hua^1,2^, Tsung-Chieh Yao^1,5^, Li-Chen Chen^1,5^, and Ming-Han Tsai^1,2^*, or Jing-Long Huang^1,5^*

Supplement Figure:

Comparing serum ferritin level between infants with or without ever having infectious diseases during infancy. Values represent means ± SD. Analysis by student t test (P = 0.03). LRTI: low respiratory tract infection; AOM: acute otitis media; UTI: urinary tract infection; AGE: acute gastroenteritis
